# Supplementary material for: Discoidin Domain Receptor 1 Regulates Runx2 during Osteogenesis of Osteoblasts and Promotes Bone Ossification via Phosphorylation of p38
Source: Int J Mol Sci. 2020 Sep 29;21(19):7210. doi: 10.3390/ijms21197210 (PMC7582985; doi:10.3390/ijms21197210)
Supplement: Supplementary file 1 [file ijms-21-07210-s001.pdf]

Discoidin Domain Receptor 1 Regulates Runx2 during Osteogenesis of Osteoblasts and Promotes Bone Ossification via Phosphorylation of p38

Liang-Yin Chou, Chung-Hwan Chen, Shu-Chun Chuang, Tsung-Lin Cheng, Yi-Hsiung Lin, Hsin-Chiao Chou, Yin-Chih Fu, Yan-Hsiung Wang and Chau-Zen Wang

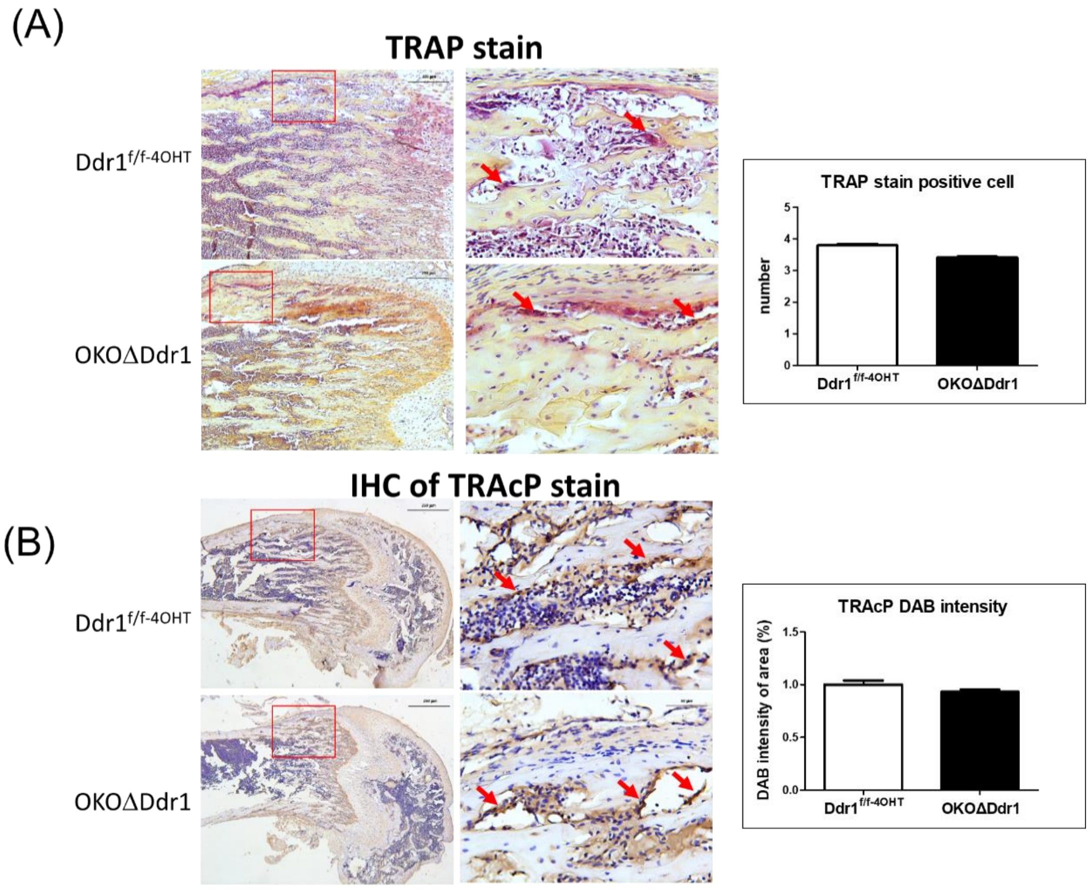

Figure 1. Knockout *Ddr1* in osteoblasts have no influence the osteoclasts. (**A**) TRAP staining the osteoclasts at 4-week-old mice femur. The magnifications were 100× and 400×, and the scale bars were 250 and 50 μm. Red arrow: TRAP positive cells. Quantification of TRAP positive cell in area. (**B**) IHC of TRAcP which was an osteoclast (OC) marker at 4-week-old femur mice. Red arrow: TRAcP positive cells. Quantification of TRAP intensity in area. Each groups more $n = 8$.
